# Retraction Note: Antidiabetic, antioxidant, antihyperlipidemic effect of extract of *Euryale ferox salisb*. With enhanced histopathology of pancreas, liver and kidney in streptozotocin induced diabetic rats

**DOI:** 10.1186/s12906-023-04064-y

**Published:** 2023-07-01

**Authors:** Danish Ahmed, Vikas Kumar, Amita Verma, Girija Shankar Shukla, Manju Sharma

**Affiliations:** 1 Department of Pharmaceutical Sciences, Faculty of Health Sciences, Sam Higginbottom Institute of Agriculture, Technology and Sciences (SHIATS)-Deemed University, Allahabad, India; 2 Department of Pharmacology, Faculty of Pharmacy, Jamia Hamdard, New Delhi, India; 3 Faculty of Health Sciences, Sam Higginbottom Institute of Agriculture, Technology and Sciences (SHIATS)-Deemed University, Allahabad, India; 4 Department of Pharmacology, Hamdard Institute of Medical Sciences and Research (HIMSR), Jamia Hamdard, New Delhi, India

**Retraction Note: BMC Complement Med Ther 4, 315 (2015)**

10.1186/s40064-015-1059-7

The Editor has retracted this article. Concerns were raised about a number of the images presented in Figs. 2, 10 and 11. The authors provided raw data; however, as there were inconsistencies in these data the Editor no longer has confidence in the results and conclusions presented. In addition, the authors have not provided evidence of appropriate ethical oversight of this study. Vikas Kumar disagrees with this retraction. Danish Ahmed, Amita Verma, Girja Shankar Shukla and Manju Sharma have not responded to correspondence from the Editor about this retraction.

